# Therapeutic effects of exendin‐4 on spinal cord injury via restoring autophagy function and decreasing necroptosis in neuron

**DOI:** 10.1111/cns.14835

**Published:** 2024-07-14

**Authors:** Xiao Gao, Qu‐Peng Li, Jing‐Ru Hao, Kai Sun, Hu Feng, Kai‐Jin Guo, Can Gao

**Affiliations:** ^1^ Nanjing Medical University Nanjing China; ^2^ Department of Orthopedics The Affiliated Hospital of Xuzhou Medical University, Xuzhou Medical University Xuzhou China; ^3^ NMPA Key Laboratory for Research and Evaluation of Narcotic and Psychotropic Drugs, Jiangsu Province Key Laboratory of Anesthesiology, Jiangsu Province Key Laboratory of Anesthesia and Analgesia Application Xuzhou Medical University Xuzhou China

**Keywords:** autophagy flux, exendin‐4, hemisection, necroptosis, SHSY5Y cell, spinal cord injury

## Abstract

**Aims:**

Necroptosis is one of programmed death that may aggravate spinal cord injury (SCI). We aimed to investigate the effect and mechanism of exendin‐4 (EX‐4) on the recovery of motor function and necroptosis after SCI.

**Methods:**

The SD rats with left hemisection in the T10 spinal cord as SCI model were used. The behavior tests were measured within 4 weeks. The effects of EX‐4 on necroptosis‐associated proteins and autophagy flux were explored. In addition, the SHSY5Y cell model was introduced to explore the direct effect of EX‐4 on neurons. The effect of lysosome was explored using mTOR activator and AO staining.

**Results:**

EX‐4 could improve motor function and limb strength, promote the recovery of autophagy flux, and accelerate the degradation of necroptosis‐related protein at 3 d after injury in rats. EX‐4 reduced lysosome membrane permeability, promoted the recovery of lysosome function and autophagy flux, and accelerated the degradation of necroptosis‐related proteins by inhibiting the phosphorylation level of mTOR in the SHSY5Y cell model.

**Conclusion:**

Our results demonstrated that EX‐4 may improve motor function after SCI via inhibiting mTOR phosphorylation level and accelerating the degradation of necroptosis‐related proteins in neurons. Our findings may provide new therapeutic targets for clinical treatment after SCI.

## Graphical abstract

This study showed that the expression of necroptosis‐related proteins increased after spinal cord injury (SCI). Exendin‐4 (EX‐4) played a therapeutic role via promoting the recovery of lysosome function and autophagy flux and accelerating the degradation of necroptosis‐related proteins by inhibiting the phosphorylation level of mTOR in neurons. This finding may provide a new therapeutic target in clinical treatment after SCI.

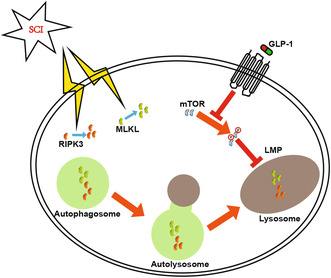

## 1 INTRODUCTION

Spinal cord injury (SCI) refers to injuries induced by external direct or indirect factors. It leads to various motor, sensory, and sphincter dysfunction, muscular dystonia, and force reflex changes in the corresponding segments. Traffic injury, low injury, and high injury are the common causes of SCI and the incidence rate from different ages shows an upward trend over time.^1^, ^2^ Usually, surgery is used to reestablish the stability of the spine and relieve pressure on the spinal cord. In nonsurgical therapy, minocycline, riluzole, granulocyte colony‐stimulating factor, and anti‐NogoA antibody have been applied in preclinical research.^3^ However, the therapeutic effect on SCI is still limited.

There are two processes in the pathophysiology of spinal cord injury: primary injury and secondary injury.^3^ Primary injury refers to the mechanical fracture or displacement of the spine caused by external trauma and results in the compression or transection of the spinal cord. Secondary injury refers to a series of cascade reactions including hemorrhage, edema, axon and neuron necrosis, and demyelination, followed by cyst formation and infarction after SCI.^4^, ^5^, ^6^ Typically, the secondary injury shows more serious damage than the primary injury due to the cascade reaction. Therefore, it is necessary to carry out certain intervention measures for this cascade reaction to protect the spinal cord.

Necroptosis, a kind of programmed cell death, is regulated by receptor‐interacting protein kinase 1(RIPK1), RIPK3, and mixed lineage kinase domain‐like protein(MLKL).^7^ Morphologically, necroptosis is characterized by the loss of plasma membrane integrity, translucent cytoplasm, and mitochondrial swelling.^8^ Mechanistically, necroptosis is mainly induced by the interaction of the death receptor family and its agonists (TNF, FasL, TRAIL).^9^ The phosphorylation of RIPK3 induced by the recruitment of RIPK1 activates MLKL, leading to the fragmentation of cell membrane and cytoplasmic content.^10^ Previous studies showed that the expression of RIPK3 in the spinal cord was increased after SCI.^11^, ^12^

Autophagy is a complex molecular pathway that transports intracellular components to the lysosomes for degradation and recovery. At low pH, lysosomes show activity with more than 50 different hydrolases.^13^ Lysosome permeabilization (LMP) is the damage of the lysosomal membrane, leading to the release of lysosomal contents to the cytoplasm and the accumulation of undegraded substances.^14^ Previous studies showed that the autophagy initiated with the inflammatory reaction after SCI and the change of lysosomal membrane permeability was the main cause of the loss of lysosomal function.^15^, ^16^ The lysosome is the degradation site of necroptosis‐related proteins.^11^ The accumulation of necroptosis‐related proteins causes cell death due to the damage of lysosomal function after SCI.

Exendin‐4 (EX‐4), an artificial synthesis of glucagon‐like peptide analogs 1 (GLP‐1), has effects on heart protection, reducing inflammation and inhibiting apoptosis.^17^ The receptor of GLP‐1 exists on the nerve cells of the spinal cord. EX‐4 has the ability to cross the blood–spinal cord barrier, so it can act on the spinal cord directly and protect nerve cells.^18^, ^19^ Research has shown that EX‐4 can promote the restoration of mitochondrial function and clear damaged mitochondria in autophagy, which is of great significance for improving diseases such as neuronal injury.^20^ EX‐4 can promote the recovery of autophagy and inhibit apoptosis to promote the recovery of motor function after SCI.^18^, ^21^ Recent study has shown that EX‐4 inhibited the necroptosis of muscle fibers in polymyositis.^22^ However, the effect and mechanism of EX‐4 on necroptosis of nerve cells after SCI is not clear.

Here, we tested whether EX‐4 could promote the recovery of motor function after SCI by promoting the recovery of autophagy and accelerating the degradation of necroptosis‐related proteins.

## 2 MATERIALS AND METHODS

### 2.1 Animals

All animal experiments were approved by the Animal Ethics Committee of Xuzhou Medical University (approval NO.L20210226440) on March 1, 2021. Female rats are used to be the experiment model because of a higher incidence of urinary retention in male rats after SCI. Sixty SPF female SD rats (weighing 200–220 g and aged 6–8 weeks) were obtained from the Experiment Animal Center of Xuzhou Medical University. The rats were feeded in the Animal Experiment Room of the Key Laboratory of Anesthesiology at a controlled temperature of 22°C with lights on from 8 am to 8 pm.

### 2.2 Rat model of spinal cord injury and drug injection

The rats were anesthetized with 30 mg/kg pentobarbital sodium (i.p., P3761, Sigma) in intraperitoneal injection and the dorsal hair was shaved around the T10 level. The skin was disinfected with iodophor and 75% alcohol was sprayed in the air. A 3 cm longitudinal incision in the median was cut with a T10 scalpel and muscle and fascia were separated with the curved ophthalmic scissors to expose the T9‐T10 level spinal cord. The tissue attached to the T10 lamina was cleaned up with the scalpel. The T10 pedicel was cut with straight ophthalmic scissors to expose the T10 spinal cord and the exposure area was enlarged with the microvascular clamp. A left hemisection of the T10 spinal cord was cut laterally three times sticking to the central vein using the scalpel (RWD, S3007‐12) with a blade width of 1.5 mm.^23^ The gelatin sponge was placed on the notch to absorb the exuded blood and the incision was sutured in layers. The bladders of unable spontaneous voiding rats were squeezed with Crede's maneuver.

The rats were pretreated with EX‐4 (4 μg/2 mL in NS, i.p.) 2 days before the surgery. After the surgery, the rats were treated at 0, 1, 2, 3, and 5 days post‐injury.

### 2.3 Behavioral assessment

The Basso, Beattie, and Bresnahan (BBB) scoring method^24^ and horizontal ladder^25^ were used to measure the hind limb motor function, and the inclined plane test^26^ and rotarod^27^ to measure the hind limb forces on days 1, 3, 5, 7, 10, 14, 21, and 28 after the surgery to evaluate the rat functional recovery.

BBB scores were used to evaluate the sequential locomotor recovery of the left hind limb, which ranged from 0 to 21, with 0 indicating paralysis and 21 indicating normal movements in a square black box with 1m^2^ The movement of the hind limbs of the rats for 20 s was observed and the score was recorded. All were repeated three times.

The side walls of the horizontal ladder (150 cm long, stick of 4 mm diameter, irregularly spaced horizontally by 2.4 cm) were made of transparent acrylic plates to observe the rat hind limb motion. Train the rats beforehand for 3 days before the surgery. The score ranged from 0 to 7 according to Andrew R et al.(2021) was modified to fit the actual situation: (0) Unable to cross the ladder; (1) Cross the ladder only by the forepaws or the ratio of the right hind limb grasping the stick was below 25%; (2) Cross the ladder and the ratio of the right hind limb over 25%; (3) Cross the ladder and the ratio of both the hind limb over 25%; (4) Cross the ladder and the ratio of the right hind limb over 50% which the left hind limb over 25%; (5) Cross the ladder and the ratio of the both hind limb over 50%; (6) Cross the ladder and the ratio of the right hind limb over 90% which the left hind limb over 50%; (7) Cross the ladder and the ratio of the both hind limb over 90%. All rats were pretraining on the horizontal ladder before the surgery.

The inclined plane test was performed on a plank of wood with a rubber mat. Maximum angle was recorded when the rat stayed for at least 5 s and repeated three times in 5 min intervals. The rats were trained at 10c/min for 5 min twice with the rotarod (ZH300) before the surgery. The rotarod was accelerated from 5c/min and the retainment time of rats was measured. The timeline of behavior tests and drug injections was shown.

### 2.4 Cell culture

The SHSY5Y cell line was gifted by Jiangsu Province Key Laboratory of Anesthesiology. The SHSY5Y cells were cultured in Dulbecco's Modified Eagle's Medium/Nutrient Mixture F‐12 Ham (D6421, sigma), which contained 10% fetal bovine serum (D8437‐500 ML, Sigma, Irvine), 100 U/mL penicillin, and 100 μg/mL streptomycin (C0222, Beyotime). Then, they were incubated at 5% CO_2_ and 37°C.

### 2.5 Western blotting

Rats were anesthetized with 40 mg/kg sodium pentobarbital and killed by decapitation. A 5 mm segment of tissue containing the hemisection was lysed in lysed buffer (Beyotime) with a 1 mM protease inhibitor cocktail. Disrupted tissues were centrifuged at 12000 rpm for 15 min at 4°C to obtain the supernatant. The amount of protein was determined using a BCA protein assay (Cat# P0011, Beyotime). Samples soaked in the solution (62.5 mM Tris–HCl, 25% glycerol, 2% SDS, 0.01% bromophenol blue) were incubated at 95°C for 5 min prior, and loaded into 6%–15%Tris‐gly gels. The gels were electrophoresed at 120 V for 90 min and transferred to a 0.45 μm PVDF (Cat#10600023, cytiva) membrane at 100 V for 90 min. Membrane was blocked with 5% nonfat milk in 0.2 mM TBST for 2 h at RT, and primary antibodies (mouse anti‐RIPK3, 1:3000, Cat#sc‐374,639, Santa; mouse anti‐MLKL, 1:3000, Cat# sc‐293,201, Santa; rabbit anti‐mTOR, Cat# A2445, ABclonal; rabbit anti‐p‐mTOR, 1:2000, Cat# AP0115ABclonal; rabbit anti‐LC3,1:3000, Cat# 3868, Cell Signaling; mouse anti‐GAPDH,1:6000, Cat# ABL1020, Abbkine; mouse anti‐p62,1:3000, Cat#sc‐28,359, Santa) in 1% BSA (TBST) incubated overnight at 4°C. Wash the membrane for 3 × 10 min, and the HRP secondary antibody (HRP‐labeled Goat Anti‐Mouse, 1:6000, Cat#A0216, Beyotime; HRP‐labeled Goat Anti‐Rabbit,1:6000, Cat#A0208, Beyotime) in 1%BSA were incubated for 2 h at RT. Wash the membrane again for 3 × 10 min. The membrane was visualized by ChemiDoc (Alliance Q9) and analyzed by Image J (Fiji Image J 1.53 t).

### 2.6 Immunofluorescent staining

Rats were anesthetized with 40 mg/kg sodium pentobarbital (i.p.) and the heart was exposed. Use normal saline to flush the blood and the 4% FPA to perfuse the tissue. The spinal cord was post‐fixed for 12 h at 4°C, and soaked in 30% sucrose for 5d. The dehydrated spinal cord was cut into 30 μm sections on Leica cryomicrotome (Leica CM1860). The section was washed for 3 × 5 min and incubated in 0.4% PBST with 10% donkey serum for 30 min. The primary antibody (mouse anti‐RIPK3, 1:200, Cat# sc‐374,639, Santa; rabbit anti‐NeuN, 1:400, Cat# 24307, Cell Signaling; rabbit anti‐GFAP, 1:400, Cat# 80788, Cell Signaling; rabbit anti‐iba1, 1:200, Cat# ab178846, abcam; mouse anti‐MLKL, 1:200, Cat# sc‐293,201; mouse anti‐p62, 1:200, Cat# sc‐28,359, Santa; rabbit anti‐LAMP1, Cat# A16894, ABclonal) was incubated overnight at 4°C. Then, the sections were washed for 3 × 5 min and the secondary antibody (Donkey anti‐Rabbit, Alexa Fluor 488, 1:400, Cat# A21206, l:400, ThermoFisher; Donkey anti‐Rabbit, Alexa Fluor 594, 1:400, Cat# A21207, ThermoFisher; Donkey anti‐Mouse, Alexa Fluor 488, 1:400, Cat# A32766, ThermoFisher; Donkey anti‐Mouse, Alexa Fluor 594, 1:400, Cat# A21203, l:400) was incubated for 2 h at RT in the dark. Wash the sections again for 3 × 5 min. After the sections were dried, the DAPI (Cat# 0100–20, Southern Biotech) was dropped on the sections and the coverslip was mounted on glass microscopy slides. Images were acquired with an Olympus FV1000 camera (Olympus Corporation) and a confocal laser scanning microscope (CLSM, Leica STELLARIS 5).

### 2.7 AO staining

AO uptake assay was used to detect the severity of lysosomal membrane damage in red fluorescence.^28^ The SHSY5Y cells were cultured in a confocal dish for 24 h and were pretreated with or without EX‐4 or MHY for 4 h. Then, the cells were treated with 200 μM H_2_O_2_ for 6 h. Clean culture dishes with PBS for 3 × 5 min and the cells were incubated with 4 μM AO dye solution at 37°C for 15 min. Observe the image under Olympus microscope (FV1000) with wavelength 488/594 nm.

### 2.8 RT‐PCR assay

SHSY5Y cells were incubated in six‐well plate with 4 × 10^5^ cells/well for 24 h, pretreated with or without EX‐4 for 6 h, and treated with 200 μM H_2_O_2_ for 6 h. The cells were washed twice with PBS and placed with 1 mL trizol/well at RT for 5 min. The lysate was piped into a 1.5 mL ribozyme‐free EP tube, and then 200 μL chloroform was added. The mixtures were centrifuged at 12000 *g* for 15 min. The supernatant was sucked into a new 1.5 mL ribozyme‐free EP tube and 500 μL isopropanol was added. Mixed them upside and down and placed them at RT for 5 min. The mixtures were centrifuged at 12000 *g* for 10 min and the supernatants were discarded. The precipitates were cleaned with 75% ethanol and shook them gently. The EP tubes were centrifuged at 1200 *g* for 5 min and dry them for 10 min. Dissolve RNA with 50 μL DEPC per tube and boiled them at 50°C for 10 min. The RNA concentration was measured on the RNA instrument (ProFlex PCR System, Thermal Cycler). The gDNA wiperMIX was used to remove DNA with the RNA 900 ng/tube. Use 20 μL HiScriptll qRT SuperMIX II system (50°C, 15 min; 85°C, 5 s) to reverse mRNA to cDNA. The 20 μL PCR reaction system included 10 μL 2 × HiFiTaq PCR Star Mix (GenStar), 0.8 μL forward and reverse primers (10 μM), 4 μL cDNA and 5.2 μL DEPC water. The program was used on the Real‐Time PCR System (Quant Studio™ 7 Flex) for all genes and included 30s predenaturation cycle at 95°C and 40 PCR cycles (95°C, 10s; 60°C, 30s). All data calculations were performed using ΔΔ CT value.^29^ Table 1 lists the primer sequences.

**TABLE 1:** Primer sequences in RT‐PCR assay.

Gene	Primers
RIPK3	F:5′‐ AATTCGTGCTGCGCCTAGAAG‐3′
	R:5′‐TCGTGCAGGTAAAACATCCCA‐3′
MLKL	F:5′‐ AGGAGGCTAATGGGGAGATAGA‐3′
	R:5′‐TGGCTTGCTGTTAGAAACCTG‐3′
mTOR	F:5′‐GCAGATTTGCCAACTATCTTCGG‐3′
	R:5′‐CAGCGGTAAAAGTGTCCCCTG‐3′
GAPDH	F:5′‐CAATGACCCCTTCATTGACC‐3′
	R:5′‐TTGATTTTGGAGGGATCTCG‐3′

### 2.9 CCK8

CCK8 is used to detect the cell activity after adding H_2_O_2_ or drug.^30^ Cells were plated on 96‐well plate with 10,000 cells/well for 24 h, pretreated with or without EX‐4 for 6 h, and treated with 200 μM H_2_O_2_ for 6 h. The SHSY5Y cells were incubated with 10% CCK8 solution at 37°C for 1 h and the absorbance at 450 nm wavelength was measured on a microplate reader (Nanodrop2000).

### 2.10 Statistical analyses

All data analyses were performed on GraphPad Prism 9.0. All data are presented as mean ± standard error of mean (SEM). The Shapiro–Wilk test was used to test for normality. The data between the two groups were compared using the two‐tailed unpaired sample *t*‐test. The data between the multiple groups were compared using one‐way or two‐way ANOVA and Bonferroni was performed to test inspection level *α* = 0.05 and *p* < 0.05 showed the difference was statistically significant.

## 3 RESULTS

### 3.1 EX‐4 improves behavioral scores and attenuates deterioration of the spinal cord after SCI in rats

**FIGURE 1:**
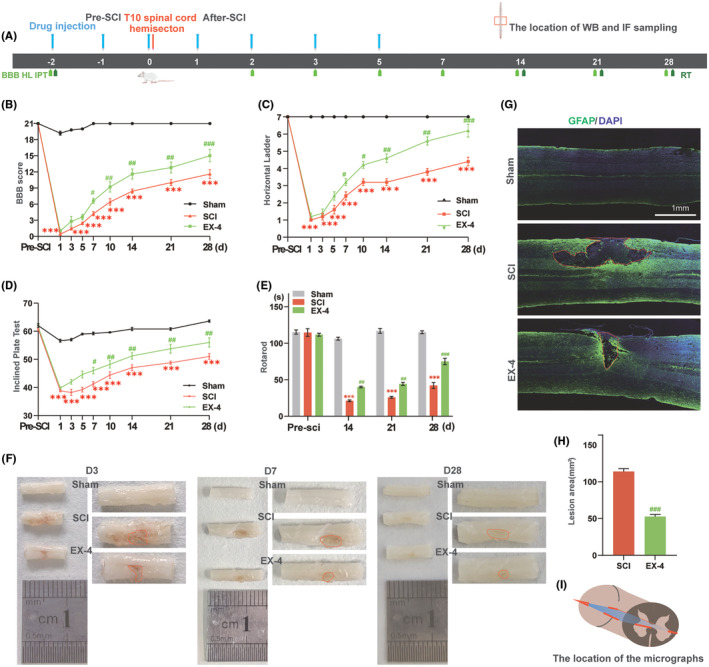
EX‐4 improves behavioral scores and attenuates deterioration of the spinal cord after SCI in rats. (A) The flowchart of the experiment. (B) BBB scores showed spontaneous recovery over time and the rats treated by EX‐4 had significantly higher BBB scores than SCI group, which showed statistical significance from day 7 to 28. (C) The horizontal ladder scores showed significantly that the recovery of the hind limbs in EX‐4 group was better compared with SCI group. (D) The inclined plate test scores of EX‐4 group were significantly better than SCI group. (E) The rotarod time of Sham, SCI, and EX‐4 groups from pre‐SCI to 28 days post‐injury showed the rats in EX‐4 group stayed longer than the SCI group from day 14. *n* = 5 for Sham, SCI, and EX‐4 groups. (F) The images of T10 spinal cord were shown in different groups at D3, D7, and D28. The red circles are unhealed incisions. Compared with SCI group, the area was significantly less in EX‐4 group. (G) The lesion cavity on the area of DAPI‐position without GFAP‐position at 28 days post‐injury. Coronal plane. Scale bars: 1 mm. (H) The area of the lesion cavity in SCI and EX‐4 group. Compared with SCI group, the area was significantly less in EX‐4 group. *n* = 3 for SCI and EX‐4 groups. (I)The schematic image (Red box) indicates the location of the micrographs. The data are shown as mean ± SEM., ***p < 0.001 versus the corresponding Sham group; ^#^ *p* < 0.05, ^##^ *p* < 0.01, ^###^ *p* < 0.001 versus The corresponding SCI group.

We first tested whether EX‐4 could rescue the motor function after SCI in rats. Behavioral observation and anatomical observation were used as evaluation indexes. BBB score, horizontal ladder, inclined plane test, and rotarod were used to evaluate the functional recovery after injury for 4 weeks after saline or EX‐4 injection (Figure 1A). Animals in EX‐4 groups showed spontaneous recovery over time and higher BBB scores than SCI group, which showed statistical significance from days 7 to 28 (Figure 1B). A horizontal ladder was performed to evaluate walking recovery and coordinated locomotion. Horizontal ladder score showed that the recovery of the hind limbs in EX‐4 group was significantly better compared with SCI group (Figure 1C). The results of BBB score and horizontal ladder confirmed that EX‐4 ameliorated the hind limb motor function after the hemisection. In the inclined plate test, the scores of EX‐4 group were significantly better than SCI group (Figure 1D). In the rotarod, the rats in EX‐4 group stayed longer than the SCI group from day 14 (Figure 1E). The results of the inclined plate test and rotarod demonstrated that the hind limb strength of rats after SCI was improved by EX‐4. These behavioral results indicated that EX‐4 could promote the recovery of hind limb motor function and strength after spinal cord hemisection.

The spinal cord images and GFAP IF staining were used to evaluate the lesion area in different groups. The spinal cord image was obtained after the tissue perfusion with 4% PFA at days 3, 7, and 28. The lesion area of the T10 spinal cord began smaller over time and EX‐4 promoted the healing of incision by assessing the areas in the red dashed boxes (Figure 1F). GFAP and DAPI in the coronal sections 28 days post‐injury were observed with Immunofluorescent staining to evaluate the area of the cystic cavity. The DAPI+ GFAP region in the solid red frame was the T10 spinal coronal capsule. Compared with SCI group, the area of EX‐4 group was significantly reduced (Figure 1G,H). Interestingly, GFAP staining on day 28 revealed that the area of the cystic cavity was significantly larger than the gross spinal cord image. The schematic image (Red box) indicates the location of the micrographs (Figure 1I). These anatomy results demonstrated that EX‐4 can attenuate the expansion of the lesion area after SCI.

### 3.2 Necroptosis is ubiquitously expressed in spinal cord neural cells after SCI in rats

**FIGURE 2:**
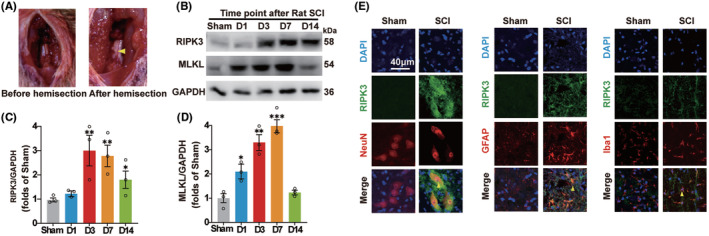
Necroptosis is ubiquitously expressed in spinal cord neural cells after SCI in rats. (A) The injury image of T10 spinal cord hemisection (The yellow arrowhead). (B) The expressions of RIPK3 and MLKL after SCI at different time points. (C,D) The quantification of RIPK3 and MLKL after SCI. The results showed that the expression of RIPK3 was increased significantly at day 3 and the expression of MLKL was increased from day 1 and reached the peak level at day 7 in the spinal cord hemisection tissue. (E) Double staining of RIPK3 with NeuN, GFAP, and Iba1 in Sham and SCI groups 3 days after injury. The yellow arrows indicated RIPK3 expression in neurons, astrocytes, and microglia. Scale bars: 40 μm. *n* = 3 for all groups. All data are shown as mean ± SEM. *p < 0.05, **p < 0.01, ***p < 0.001 versus the corresponding Sham group.

To detect whether necroptosis occurred after SCI, The T10 spinal cord left hemisection was performed by the scalpel (Figure 2A). Immunoblotting was performed to detect the level of necroptosis‐associated protein, including RIPK3 and MLKL, during 2 weeks within the spinal cord hemisection around 1 cm in diameter in the rat T10 spinal cord segment (Figure 2B). The results showed that the expression of RIPK3 was increased significantly at day 3 and the expression of MLKL was increased from day 1 and reached the peak level at day 7 in the spinal cord hemisection tissue (Figure 2C,D). NeuN serves as a neuronal marker, GFAP is an astrocytic marker, and Iba1 is a marker for microglial cells. The colocalization of RIPK3 and nerve cell markers was detected at day 3 after SCI. The RIPK3 expressed significantly in neurons, astrocytes, and microglia (Figure 2E). The results verified that necroptosis is ubiquitously expressed in spinal cord neural cells after SCI.

### 3.3 EX‐4 attenuates the necroptosis expression in neurons after SCI

**FIGURE 3:**
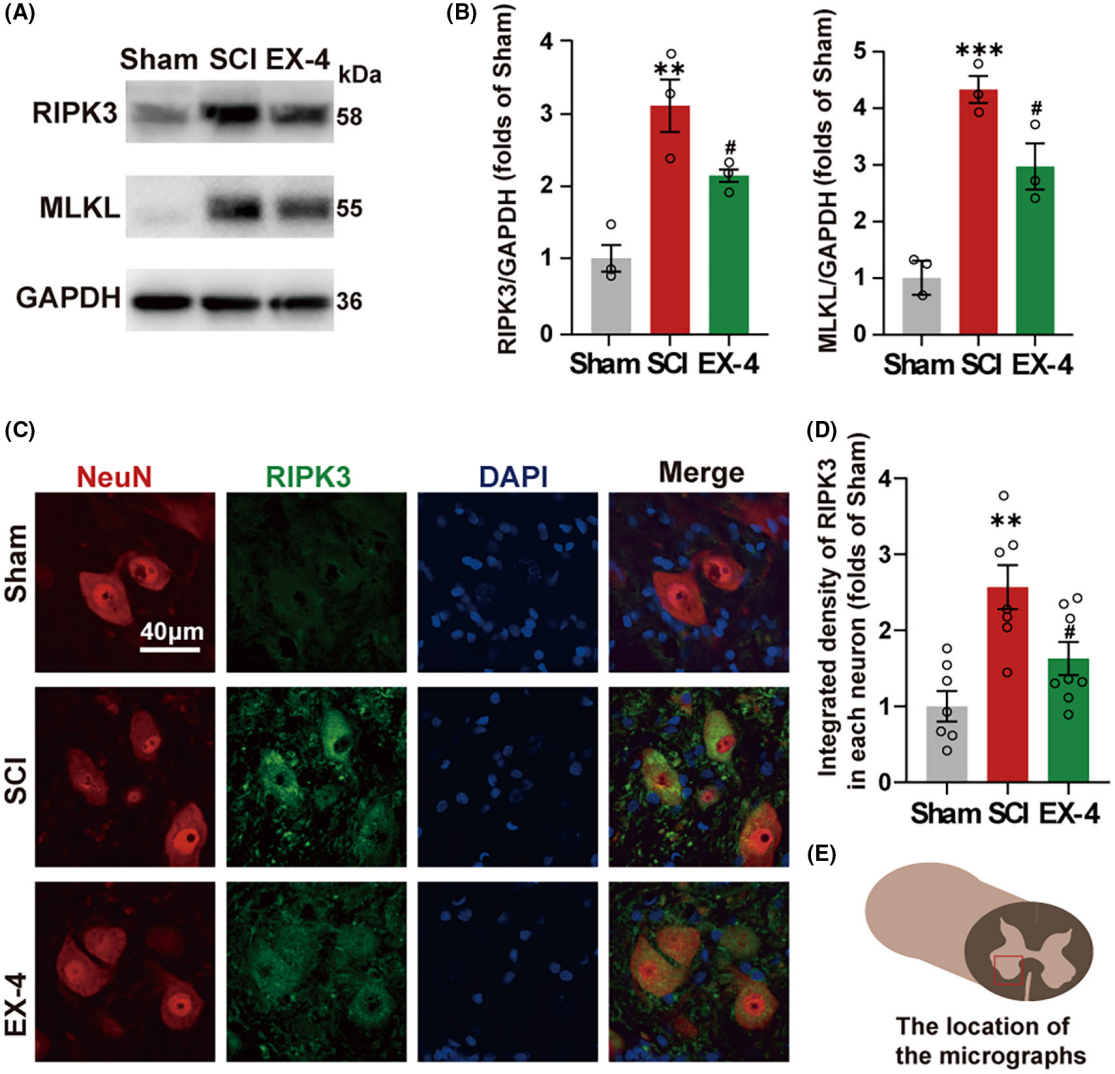
EX‐4 attenuates the necroptosis expression in neurons after SCI. (A)The immunoblot expressions of RIPK3 and MLKL in Sham, SCI, and EX‐4 groups. (B) The quantification of RIPK3 and MLKL. The expressions of RIPK3 and MLKL were higher in SCI group than Sham group. *n* = 3 for all groups. (C) Immunofluorescent staining for RIPK3 and NeuN colocalization in ventral motor neurons of T10 spinal cord. Scale bar = 40 μm. (D) The quantification of the average density of RIPK3 in neurons. These results suggested that EX‐4 can attenuate the necroptosis in the spinal cord after the surgery. *n* = 7 for Sham group and SCI group. *n* = 8 for EX‐4 group. (E) The schematic image (Red box) indicates the location of the micrographs. All data are shown as mean ± SEM. **p < 0.01, ***p < 0.001 versus the corresponding Sham group; ^#^ *p* < 0.05 versus the corresponding SCI group.

Next, we determined whether EX‐4 had a therapeutic effect on necroptosis after the SCI. The tissue of spinal cord injury at day 3 in Sham, SCI, and EX‐4 groups were used to perform immunoblot analysis (Figure 3A). The expressions of RIPK3 and MLKL were higher in SCI group than Sham group (Figure 3B). Compared with the SCI group, both expressions of RIPK3 and MLKL were reduced in EX‐4 treatment group (Figure 3B). Immunofluorescent staining revealed that RIPK3 was expressed within the ventral neurons in the lesions (Figure 3C **)**. The expression of RIPK3 in neurons was reversed significantly after EX‐4 treatment compared with SCI group (Figure 3D). These results suggested that EX‐4 could attenuate the necroptosis in the spinal cord after SCI. EX‐4 may promote the recovery of hind limb motor function by rescuing the necroptosis of the ventral neuron after SCI.

### 3.4 EX‐4 restores the autophagy function and inhibits the process of necroptosis after SCI

**FIGURE 4:**
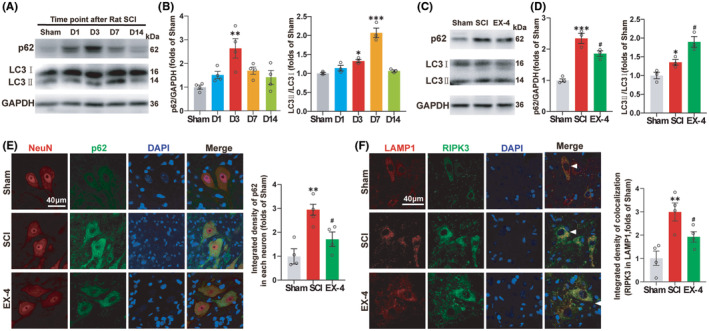
EX‐4 restores the autophagy function and inhibits the process of necroptosis after SCI. (A) The immunoblot expressions of p62 and LC3 in Con, D1, D3, D7, and D14 groups. (B) The p62 was accumulated gradually and reached the peak at day 3, while the LC3II was accumulated gradually and reached the peak at day 7. *n* = 3 for p62 of all groups. *n* = 4 for LC3 of all groups. (C) The immunoblot expression of p62 and LC3 in Sham, SCI, and EX‐4 groups. (D) The expressions of p62 and LC3 in EX‐4 group were significantly lower than SCI group at day 3. *n* = 3 for all groups. (E) Immunofluorescent staining for p62 and NeuN colocalization in ventral motor neurons of T10 spinal cord and the quantification of average density of p62 in neurons. The accumulated p62 was decreased significantly after the treatment of EX‐4 due to the recovery of autophagy flux. Scale bar = 40 μm. *n* = 4 for all groups. (F) Immunofluorescent staining for RIPK3 and LAMP1 colocalization in ventral motor neurons of T10 spinal cord and the quantification of the average density of RIPK3 on the colcocalization area of RIPK3 in LAMP1. The expression of RIPK3 in lysosome was decreased significantly after EX‐4 treatment. Scale bar = 40 μm. *n* = 4 for all groups. All data are shown as mean ± SEM. *p < 0.05, **p < 0.01, ***p < 0.001 versus the corresponding Sham group; ^#^ *p* < 0.05 versus the corresponding SCI group.

Previous study^11^ demonstrated that RIPK3 and MLKL were degraded in the lysosome and the autophagy function of lysosome degrading the protein was decreased after SCI. P62 and LC3 are reliable indicators of autophagic flux dynamics. We then analyzed the expressions of p62 and LC3 at different time points to determine the change of autophagic flux after SCI (Figure 4A). The p62 was accumulated gradually and reached the peak level at day 3, while the LC3II was accumulated gradually and reached the peak level at day 7 (Figure 4B). These results indicated that the autophagy flux was blocked and the autophagy function of the spinal cord was actually inhibited after the SCI.

Next, we tested whether EX‐4 can restore the autophagy function of the spinal cord at day 3 (Figure 4C). The results showed that the expressions of p62 and LC3 in EX‐4 group were significantly lower than SCI group at day 3 (Figure 4D). The autophagy flux and the degradation function of lysosome were restored. In addition, Immunofluorescent results of p62 in neurons showed the same trends as that observed in the western blot (Figure 4E). The accumulated p62 was decreased significantly after the treatment of EX‐4 due to the recovery of autophagy flux.

LAMP1 is a lysosomal‐associated membrane protein that is commonly used as a marker to identify lysosomes. To verify whether EX‐4 can promote the recovery of lysosome function on degrading RIPK3, Immunofluorescent was used to observe the double staining of RIPK3 with LAMP1 at day 3. The arrowheads indicated that RIPK3 was located in the lysosome. The expression of the double positive regions of RIPK3 and LAMP1 increased significantly after SCI compared to Sham group. The expression of RIPK3 in lysosome was decreased significantly after EX‐4 treatment (Figure 4F). The above results suggested that EX‐4 may promote the recovery of autophagy in spinal neurons and inhibit the process of necroptosis after SCI.

### 3.5 EX‐4 reduces the necroptosis induced by H_2_O_2_ in the SHSY5Y cell model

**FIGURE 5:**
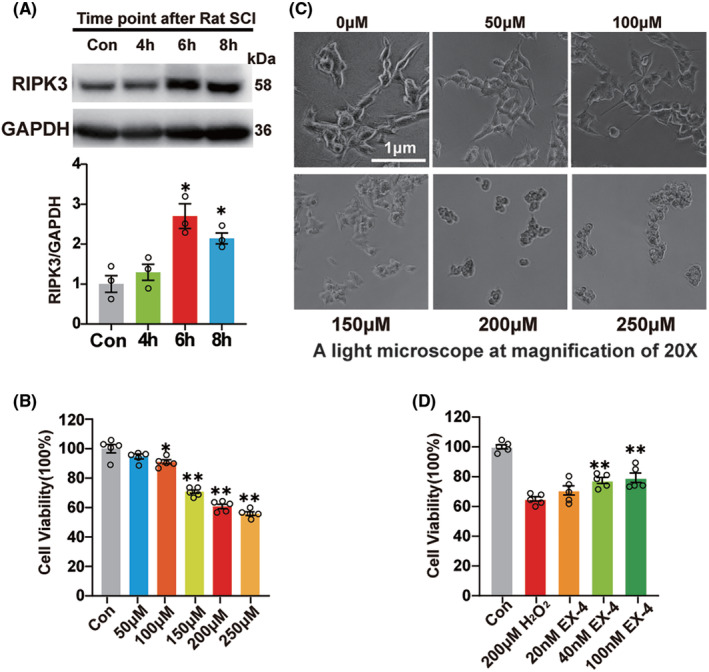
EX‐4 reduces the necroptosis induced by H_2_O_2_ in SHSY5Y cell model. (A) The immunoblot expression of RIPK3 at 4 h, 6 h, and 8 h. The highest expression was at 6 h. The data are shown as *n* = 3. (B) The SHSY5Y cells were incubated with 0, 50, 100, 150, 200, and 250 μM H_2_O_2_ for 6 h. The survival viability of SHSY5Y cells was determined by CCK8 assay. The cell activity was significantly decreased after treatment with 200 and 250 μM H_2_O_2_, with the mean value lower than 70%. *n* = 5 for all groups. (C) The injury images of SHSY5Y cells in 0, 50, 100, 150, 200, and 250 μM H_2_O_2_ for 6 h (in a light microscope of 20X). (D) The SHSY5Y cells were pretreated with 0, 20, 40, and 100 nM EX‐4 for 4 h and incubated in 200 μM H_2_O_2_ for 6 h. The survival viability of SHSY5Y cells was determined by CCK8 assay. The results showed that 40 and 100 nM EX‐4 improved the survival of SHSY5Y significantly compared to the H_2_O_2_ group. *n* = 5 for all groups. All data are shown as mean ± SEM. *p < 0.05, **p < 0.01 versus the corresponding Con group.

To further verify the mechanism of EX‐4, SHSY5Y cells were incubated with 200 μM H_2_O_2_ for 4, 6, and 8 h, respectively. Immunoblot was used to detect the expression of RIPK3 and the highest expression was observed at 6 h (Figure 5A). Then, SHSY5Y cells were treated at concentrations of 0, 50, 100, 150, 200, and 250 μM H_2_O_2_ for 6 h, and incubated with CCK8 for 1 h to detect the cell activity. The result showed that the cell activity was significantly decreased after treatment with 200 and 250 μM H_2_O_2_, with the mean value lower than 70% (Figure 5B). The SHSY5Y cells were shrunk obviously under the high concentration, so 200 μM was selected for the subsequent experiments (Figure 5C). Then the cells were pretreated with 10, 40, and 100 nM EX‐4 for 4 h, followed by 200 μM H_2_O_2_ for 6 h, and then incubated with CCK8 for 1 h. The results showed that 40 and 100 nM EX‐4 improved the survival of SHSY5Y significantly compared to the H_2_O_2_ group (Figure 5D). So 100 nM EX‐4 was selected for the subsequent experiments.

### 3.6 EX‐4 promotes autophagy and accelerates the degradation of RIPK3 and MLKL

**FIGURE 6:**
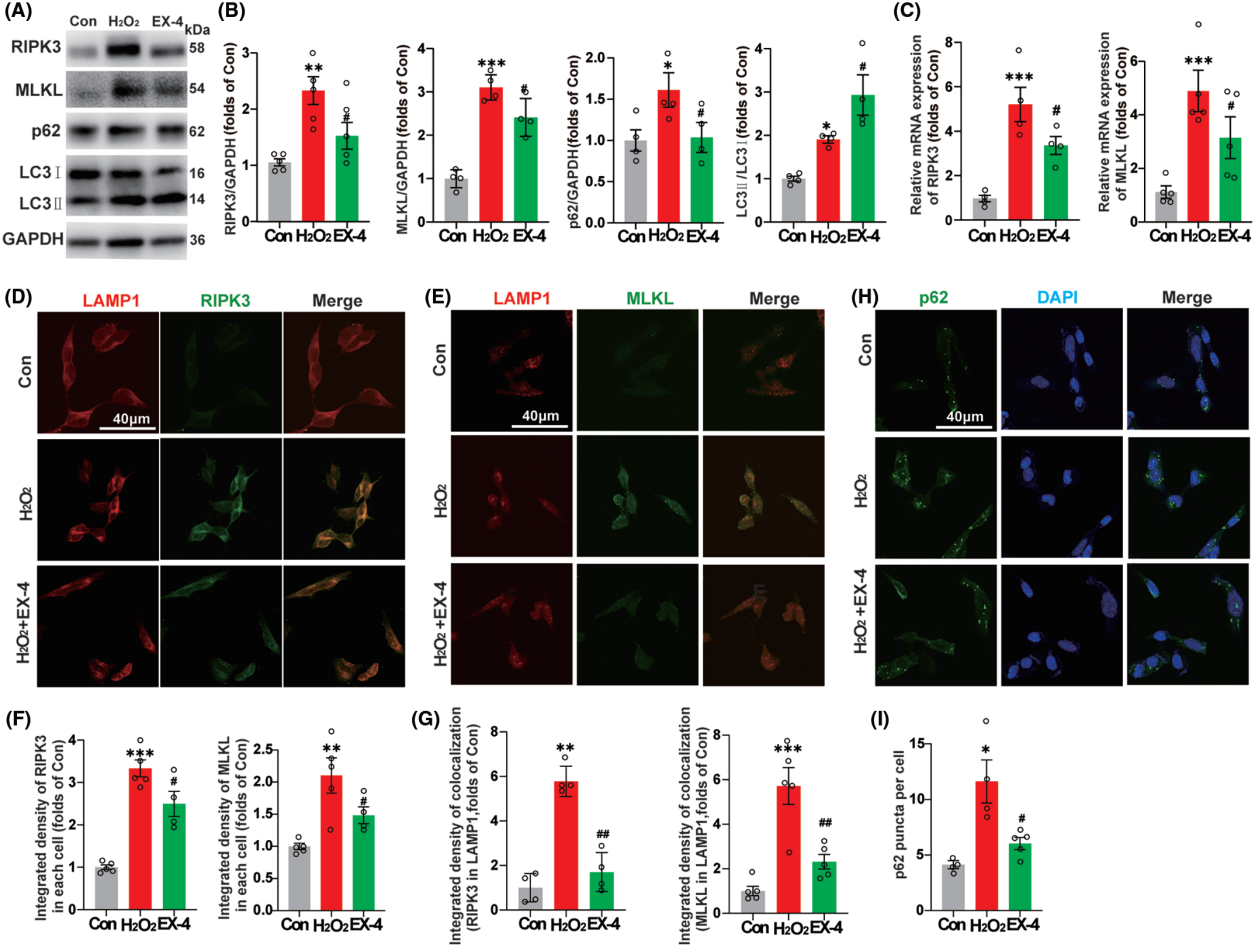
EX‐4 promotes autophagy and accelerates the degradation of RIPK3 and MLKL. The immunoblot expressions of RIPK3, MLKL, p62, and LC3 of SHSY5Y in Con, H_2_O_2_, and EX‐4 groups. (B) The quantification of Figure 6A. The expressions of RIPK3, MLKL, p62, and LC3 were consistent with those in vivo. EX‐4 attenuated the necroptosis and promoted the recovery of autophagy flux. (C) The mRNA expression of RIPK3 and MLKL of SHSY5Y cells in Con, H_2_O_2_, and EX‐4 groups. The mRNA levels of RIPK3 and MLKL in H_2_O_2_ group were significantly upregulated, and the high expression of RNA was reversed in EX‐4 group. (D) Immunofluorescent staining for LAMP1 and RIPK3 colocalization. (E) Immunofluorescent staining for LAMP1 and MLKL colocalization on SHSY5Y cells of Con, H_2_O_2_, and EX‐4 groups. Scale bar = 40 μm. (F) The quantification of the average density of RIPK3 and MLKL showed that the expression reversed significantly in EX‐4 group compared to H_2_O_2_ group. (G) The accumulation of RIPK3 and MLKL in the lysosome increased significantly in H_2_O_2_ group compared with Con group and the accumulation was decreased in EX‐4 group. (H)The p62 puncta of SHSY5Y in Con, H_2_O_2_, and EX‐4 groups. Scale bar = 40 μm. (I) The number of p62 puncta was significantly high in H_2_O_2_ group. However, EX‐4 group had a lower number than H_2_O_2_ group. *n* ≥ 4 for all groups. All data are shown as mean ± SEM. *p < 0.05, **p < 0.01, ***p < 0.001 versus the corresponding Con group; ^#^ *p* < 0.05, ^##^ *p* < 0.01 versus the corresponding H_2_O_2_ group.

To verify whether EX‐4 promoted the recovery of autophagy flux and lysosome function in the SHSY5Y cell, 200 μM H_2_O_2_ for 6 h was applied after the pretreatment with 100 μM EX‐4 for 4 h. The expressions of RIPK3, MLKL, p62, and LC3 were consistent with those in vivo. EX‐4 attenuated the necroptosis and promoted the recovery of autophagy flux (Figure 6A,B). The mRNA levels of RIPK3 and MLKL in H_2_O_2_ group were significantly upregulated, and the high expression of RNA was reversed in EX‐4 group (Figure 6C).

Next, we tested whether EX‐4 promoted the recovery of lysosome function on degrading RIPK3 and MLKL with double staining of RIPK3 and MLKL with lysosomal marker LAMP1 (Figure 6D,E). The quantification of the average density of RIPK3 and MLKL showed that the expression reversed significantly in EX‐4 group compared to H_2_O_2_ group (Figure 6F). The accumulation of RIPK3 and MLKL in the lysosome increased significantly in H_2_O_2_ group compared with Con group and the accumulation was decreased in EX‐4 group (Figure 6G). The p62 puncta of SHSY5Y was observed in Con, H_2_O_2_, and EX‐4 groups (Figure 6H). The number of p62 puncta was significantly high in H_2_O_2_ group. However, EX‐4 group had a lower number than H_2_O_2_ group (Figure 6I). These results suggested that EX‐4 promoted lysosomal recovery and autophagy, thereby enhancing the degradation of RIPK3 and MLKL in lysosomes.

### 3.7 EX‐4 attenuates necroptosis via inhibiting the mTOR phosphorylation

**FIGURE 7:**
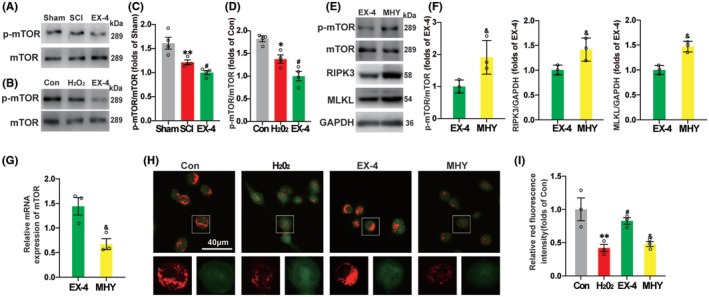
EX‐4 inhibits necroptosis by reducing phosphorylation of mTOR and recovering lysosomal function in SHSY5Y cells. (A) The immunoblot expression of p‐mTOR and mTOR of SD rats at day 3 in Sham, SCI, and EX‐4 groups. (B) The immunoblot expression of p‐mTOR and mTOR of SHSY5Y cells in Con, H_2_O_2_, and EX‐4 groups. (C,D) Compared with Sham group, the ratio of p‐mTOR and mTOR was decreased significantly. The phosphorylation level continued to decrease in EX‐4 group, compared with SCI group. *n* = 4 for all groups. (E,F) MHY1485, a mTOR agonist, upregulated mTOR phosphorylation level and the expressions of RIPK3 and MLKL compared with EX‐4 group. *n* = 3 for all groups. (G) The mRNA expression of mTOR of SHSY5Y cells in EX‐4 and MHY groups. The mRNA level of mTOR was downregulated. *n* = 3 for all groups. (H) The AO staining of SHSY5Y cells in Con, H_2_O_2_, EX‐4, and MHY groups. The decreasing of red fluorescence intensity indicated the increasing of lysosomal permeability (LMP). (I) Compared with Con group, LMP in H_2_O_2_ group was significantly increased. In EX‐4 group, lysosomal LMP decreased significantly after EX‐4 treatment. *n* = 3 for all groups. All data are shown as mean ± SEM. *p < 0.05, **p < 0.01 versus the corresponding Sham or Con group; ^#^ *p* < 0.05 versus the corresponding SCI or H_2_O_2_ group; ^&^ *p* < 0.05 versus the corresponding EX‐4 group.

Considering the activation of mammalian target of rapamycin (mTOR) signal after SCI regulates various physiological and pathological processes,^31^ we further tested the role of mTOR signaling pathway. Compared with Sham group, the ratio of p‐mTOR and mTOR was decreased significantly, which indicated that the autophagy was initiated after SCI. The phosphorylation level continued to decrease in EX‐4 group, compared with SCI group (Figure 7A,C). In the vitro model of SHSY5Y cells, the phosphorylation level was decreased significantly in EX‐4 group compared with H_2_O_2_ group (Figure 7B,D), which was consistent with the results in vivo.

MHY1485, a mTOR agonist, upregulated mTOR phosphorylation level and the expressions of RIPK3 and MLKL compared with EX‐4 group (Figure 7E,F). These results indicated that the process of necroptosis of spinal cord neurons was inhibited with EX‐4 by downregulating the phosphorylation of mTOR. The mRNA level of mTOR was downregulated, which may be related to the increased phosphorylation level of mTOR in MHY group (Figure 7G). The lysosomal membrane permeability of SHSY5Y cells was detected by AO staining. Compared with Con group, LMP in H_2_O_2_ group was significantly increased. In EX‐4 group, lysosomal LMP decreased significantly after EX‐4 treatment. The therapeutic effect of EX‐4 was inhibited after MHY administration, indicating that EX‐4 reduced LMP by inhibiting mTOR phosphorylation (Figure 7H,I). These results indicated that the recovery of lysosomal function was promoted with EX‐4 by inhibiting the phosphorylation process of mTOR and reducing LMP, which promoted the degradation of necroptosis‐related proteins and reduced their recruitment and phosphorylation level.

## 4 DISCUSSION

The results of this study showed that EX‐4 promoted the recovery of motor function after SCI, alleviated the necroptosis of ventral motor neurons, and limited the expansion of the injury area. In addition, EX‐4 promoted the recovery of lysosomal function and accelerated the degradation of necroptosis‐related proteins by inhibiting the phosphorylation of mTOR in the SHSY5Y cell model in vitro, which may be related to the decrease of lysosomal membrane permeability.

EX‐4 is the first GLP‐1 RA drug approved for marketing in the world and used in clinical treatment for type 2 diabetes for a long time with its pharmacological properties tested.^32^ Recent studies have shown that EX‐4 has potential therapeutic effects on various diseases, especially in cardiovascular disease, obesity, nonalcoholic fatty liver disease, Alzheimer's disease, kidney disease, and certain types of cancer.^33^, ^34^, ^35^ Previous studies have reported that activating GLP‐1R promoted the recovery of spinal cord in rats.^18^, ^21^, ^36^ However, the detailed mechanisms are still unknown. Our studies explored the relationship between the inhibition of mTOR phosphorylation by EX‐4 and the necroptosis of spinal cord neurons. In addition, EX‐4 regulated the gene expression of necroptosis‐related proteins and inhibited the transcription level in mRNA expression after SCI. It provided a new theoretical basis for the possibility of EX‐4 in the clinical treatment of SCI.

Autophagy is an important way of recycling the cell components after the proteins degraded through lysosomes and involved in the process of many diseases.^37^ After SCI, the autophagy process starts to absorb the broken and dead cell components.^38^ More and more evidences indicated that the activation of autophagy after SCI was beneficial to the recovery of animal motor function.^39^ However, autophagic flux is inhibited after SCI and lysosomes are difficult to play a normal role, which leads to the accumulation of necroptosis‐related proteins and cell death.^11^ GLP‐1 can promote autophagy. Studies have shown that GLP‐1 analogs can significantly induce autophagy in cells. Liraglutide inhibits cell growth in a dose‐dependent manner in endometrial cancer cells and significantly induces autophagy.^40^ Autophagy can improve cell metabolism. By promoting autophagy, GLP‐1 analogs may improve the cellular metabolic state, such as restoring mitochondrial function and increasing the efficiency of cells in utilizing energy. GLP‐1 and autophagy may have a synergistic effect on protecting cells from damage and maintaining cellular homeostasis. For example, in diabetes treatment, GLP‐1 analogs alleviate damage to pancreatic beta cells by promoting autophagy and inhibiting cell apoptosis.^41^ Our studies demonstrated that the lysosomal membrane permeabilization after SCI led to the release of lysosomal contents to the cytoplasm that downregulated the degradation ability of lysosomes. In terms of the reduction of LMP with EX‐4, we speculate that it may be related to the increase of lysosomal production and faster generation of new lysosomes. This needs further investigation in the future.

Necroptosis is one of the cell‐programmed death models discovered in recent years, mainly through the TNF‐α family activating apoptosis‐related receptor,^42^ in which RIPK3 and MLKL play an important role in the recruitment and activation of it. The phosphorylation of MLKL by RIPK3 leads to necroptosis by destroying the plasma membrane and cell lysis. In our study, EX‐4 inhibited the upregulation of RIPK3 and MLKL after SCI. mTOR is a major regulator of cell growth and metabolism. Inhibition of mTOR activity can promote spinal cord repair.^43^ We found that MHY1485, a mTOR activator, inhibited the therapeutic effect of EX‐4. These results indicate that EX‐4 may promote the recovery of autophagy function by inhibiting mTOR phosphorylation.

For other cells such as astrocytes, the effect of exenatide on astrocytes mainly manifests through improving the function and metabolic status of neurons, which indirectly affects the function of astrocytes, and possibly directly binding to GLP‐1R on astrocytes to directly affect astrocytes.^44^ For oligodendrocytes, the specific action of exenatide is not yet clear, but considering its general role in the nervous system and the potential expression of GLP‐1 receptors on oligodendrocytes, exenatide may have an indirect or direct effect on oligodendrocytes. However, these speculations require further experiments and studies to verify.^45^

Traumatic SCI is one of the most difficult problems in the world. The severity of traumatic SCI mainly depends on the cascade reaction caused by the secondary injury.^3^ The vicious circle leads to the gradual expansion of the range of lesions. The damage of human physiological function becomes more and more serious. Because of the importance of the spinal cord to maintain the special functions of the human body and the nonrenewable nature of neurons,^46^ expect supporting treatment such as decompression and oxygen supply.^47^ However, conventional surgical methods have little effect. The treatment of nonsurgical treatment is drug therapy, cell transplantation, and material scaffold.^48^, ^49^ Currently, the treatment of SCI mainly includes two aspects. One is preventing further expansion of secondary injury and avoiding cell death,^50^ another one is stimulating axon regeneration and re‐establishing effective neural control function.^51^ Differ from other diseases, it is difficult to explain the cause of SCI with single or multiple pathological mechanisms. As the protagonist of the nervous system, cell status, and survival of neurons affect the motor function of bodies.

The hemisection model in the study was mainly based on the following two aspects. First, the model ensured the severity of SCI and fully damaged neurons and axons.^52^ Second, the model preserved half of the neuromotor function of rats and ensured the survival rate of rats by reducing postoperative complications. Previous studies have shown that female rats after SCI significantly reduce the occurrence of neurological injury symptoms such as urinary incontinence.^53^ Subsequently, SHSY5Y model was introduced to verify the single effect of EX‐4 on neurons and its potential mechanism. SHSY5Y cells, derived from human neuroblastoma cells, were used to simulate the effect of EX‐4 on human body. The limitation is that the effect of EX‐4 on primary neurons after injury has not been verified in vitro, and the direct effect of EX‐4 on spinal cord neurons can only be simulated in vivo.

## 5 CONCLUSION

The present study demonstrated that EX‐4 promoted functional recovery after SCI by inhibiting mTOR phosphorylation, promoting lysosomal formation, degrading RIPK3 and MLKL, and reducing lysosomal membrane permeability. Our study provided a new theoretical basis for the possibility of EX‐4 in the clinical treatment of SCI in the future.

## CONFLICT OF INTEREST STATEMENT

The authors declare no conflict of interests.

## Data Availability

The data that support the findings of this study are available from the corresponding author upon reasonable request.
